# (*E*)-2-(4-Fluoro­benzyl­idene)cyclo­octanone

**DOI:** 10.1107/S1600536808037653

**Published:** 2008-11-22

**Authors:** Yu-Lin Zhu, Min Xie, Jie Zheng, Changquan Deng

**Affiliations:** a School of Chemistry and Environment, South China Normal University, Guangzhou 510006, People’s Republic of China

## Abstract

The title compound, C_15_H_17_FO, was prepared directly from the aldol condensation of cyclo­octa­none with 4-fluoro­benz­aldehyde, catalysed by Pd(Ni,Ce) in the presence of trimethyl­silyl chloride. The eight-membered ring adopts a boat-chair conformation.

## Related literature

For related structures, see: Huang, Zhu & Pan (2004 ▶); Huang, Zhu, Pan & Wan (2004 ▶); Zhu & Pan (2004 ▶). For general background, see: Amal Raj & Raghathan (2002 ▶); Deli *et al.* (1984 ▶).
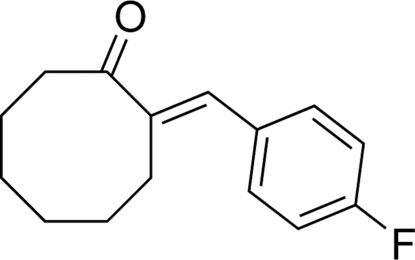


## Experimental

### 

#### Crystal data

C_15_H_17_FO
*M* *_r_* = 232.29
Orthorhombic, 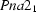

*a* = 12.0310 (2) Å
*b* = 8.6056 (1) Å
*c* = 12.2438 (2) Å
*V* = 1267.65 (3) Å^3^
*Z* = 4
Mo *K*α radiation
μ = 0.08 mm^−1^
*T* = 273 (2) K
0.20 × 0.15 × 0.10 mm

#### Data collection

Bruker APEXII CCD diffractometer
Absorption correction: multi-scan (*SADABS*; Sheldrick, 2004 ▶) *T*_min_ = 0.983, *T*_max_ = 0.998
10067 measured reflections
1584 independent reflections
1584 reflections with *I* > 2σ(*I*)
*R*_int_ = 0.036

#### Refinement

*R*[*F* ^2^ > 2σ(*F* ^2^)] = 0.037
*wR*(*F* ^2^) = 0.082
*S* = 1.07
1584 reflections
155 parameters
1 restraint
H-atom parameters constrained
Δρ_max_ = 0.11 e Å^−3^
Δρ_min_ = −0.11 e Å^−3^

### 

Data collection: *APEX2* (Bruker, 2004 ▶); cell refinement: *SAINT* (Bruker, 2004 ▶); data reduction: *SAINT*; program(s) used to solve structure: *SHELXS97* (Sheldrick, 2008 ▶); program(s) used to refine structure: *SHELXL97* (Sheldrick, 2008 ▶); molecular graphics: *SHELXTL* (Sheldrick, 2008 ▶); software used to prepare material for publication: *SHELXTL*.

## Supplementary Material

Crystal structure: contains datablocks global, I. DOI: 10.1107/S1600536808037653/rk2112sup1.cif

Structure factors: contains datablocks I. DOI: 10.1107/S1600536808037653/rk2112Isup2.hkl

Additional supplementary materials:  crystallographic information; 3D view; checkCIF report

## Figures and Tables

**Fig. 1.**
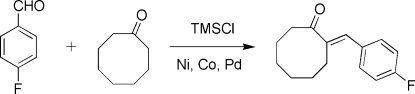
Pd(Ni, Ce)-TMSCl catalyzed synthesis of the title compound.

**Fig. 2.**
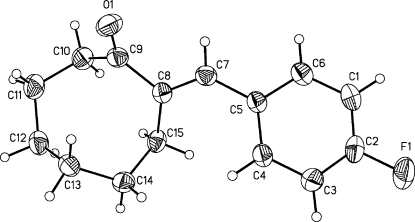
A view of the molecule of (I), with the atomic numbering scheme. Displacement ellipsoids are drawn at the 50% probability level. H atoms are shown as a small spheres of arbitrary radius.

## supplementary crystallographic information

### Comment

Arylmethyllidenecycloalkanones are a particularly important class of compounds which are used as precursors for the synthesis bioactive pyrimidine derivatives (Amal Raj & Raghathan, 2002; Deli *et al.*1984.) The aldol reaction, which is performed in the presence of strong acids, is one of the most useful reactions in organic chemistry. The reaction between cyclooctanone with 4-fluorobenzaldehyde afforded (*E*)-2-(4-fluorobenzylidene)cyclooctanone, I, (other than (2,8)-di-4-fluorobenzylidenecyclooctanone in excellent yield in the presence of Pd(Ni, Ce)-*TMS*Cl system, where *TMS*Cl is trimethylsilyl chloride, (Fig. 1) (Huang, Zhu & Pan, 2004; Huang, Zhu, Pan & Wan, 2004; Zhu & Pan, 2004). The molecule of I contains one eight-membered ring which adopts a boat-chair conformation and a phenyl ring. The boat-chair conformation is favourable for the cyclooctanone ring of I (Fig. 2). There are no unusual bond lengths and angles in the I. The C5/C7/C8/C15 torsion angle of -3.2 (3)°, to gather with C5/C7/C8/C9 torsion angle of 179.99 (17)°, describes the *E*-configuration of the molecule about the C7═C8 bond. The C7═C8 bond doesn't conjugate with C9═O1 bond due to the C7/C8/C9/O1 torsion angle has a value of -31.6 (3)° and the length of the double bonds is also normal. Similarly, the C4/C5/C7/C8 torsion angle has a value of -44.6 (3)° and the dihedral angel between the C7═C8–C5 plane with phenyl ring plane, so the C7═C8 bond do not conjugate with the phenyl ring. From the crystal packing of the title compound, the packing of molecule involves van der Waals interactions.

### Experimental

A mixture of cyclooctanone (10 mmol), 4-fluorobenzaldehyde (10 mmol), palladium (0.10 mmol), and *TMS*Cl (11 mmol) was refluxed in acetonitrile (12 ml) under 353 K for 5 h. After being cooled to room temperature, the reaction mixture was poured into water, the residue was filtration through a silica pad, and then washed twice with water, dried under vacuum to yield the products I. Single crystal of the I was obtained by slow evaporation from ethanol at room temperature.

### Refinement

The H atoms were positioned geometrically and allowed to ride on their parent atoms, with C–H = 0.93 and 0.97 Å, and *U*_iso_(H) = 1.2*U*_eq_(C).

### Crystal data

**Table:**

C_15_H_17_FO	*F*_000_ = 496
*M**_r_* = 232.29	*D*_x_ = 1.217 Mg m^−^^3^
Orthorhombic, *P**n**a*2_1_	Mo *K*α radiation λ = 0.71073 Å
Hall symbol: P 2c -2n	Cell parameters from 2166 reflections
*a* = 12.0310 (2) Å	θ = 2.9–22.2º
*b* = 8.60560 (10) Å	µ = 0.08 mm^−^^1^
*c* = 12.2438 (2) Å	*T* = 273 (2) K
*V* = 1267.65 (3) Å^3^	Block, colourless
*Z* = 4	0.20 × 0.15 × 0.10 mm

### Data collection

**Table:**

Bruker APEXII CCD diffractometer	1584 independent reflections
Radiation source: Fine-focus sealed tube	1584 reflections with *I* > 2σ(*I*)
Monochromator: Graphite	*R*_int_ = 0.036
*T* = 273(2) K	θ_max_ = 27.9º
φ and ω scans	θ_min_ = 2.9º
Absorption correction: multi-scan(SADABS; Sheldrick, 2004)	*h* = −15→12
*T*_min_ = 0.983, *T*_max_ = 0.998	*k* = −11→10
10067 measured reflections	*l* = −16→13

### Refinement

**Table:**

Refinement on *F*^2^	Hydrogen site location: Geom
Least-squares matrix: Full	H-atom parameters constrained
*R*[*F*^2^ > 2σ(*F*^2^)] = 0.037	*w* = 1/[σ^2^(*F*_o_^2^) + (0.0347*P*)^2^ + 0.02*P*] where *P* = (*F*_o_^2^ + 2*F*_c_^2^)/3
*wR*(*F*^2^) = 0.082	(Δ/σ)_max_ < 0.001
*S* = 1.07	Δρ_max_ = 0.11 e Å^−^^3^
1584 reflections	Δρ_min_ = −0.10 e Å^−^^3^
155 parameters	Extinction correction: SHELXL97 (Sheldrick, 2008), Fc^*^=kFc[1+0.001xFc^2^λ^3^/sin(2θ)]^-1/4^
1 restraint	Extinction coefficient: 0.011 (2)
Primary atom site location: Direct	Absolute structure: Since the molecule contains only light atoms, the intensities of 946 Friedels pairs were merged.
Secondary atom site location: Difmap	

### Special details

**Table:**

Geometry. All s.u.'s (except the s.u. in the dihedral angle between two l.s. planes) are estimated using the full covariance matrix. The cell s.u.'s are taken into account individually in the estimation of s.u.'s in distances, angles and torsion angles; correlations between s.u.'s in cell parameters are only used when they are defined by crystal symmetry. An approximate (isotropic) treatment of cell s.u.'s is used for estimating s.u.'s involving l.s. planes.
Refinement. Refinement of *F*^2^ against ALL reflections. The weighted *R*-factor *wR* and goodness of fit *S* are based on *F*^2^, conventional *R*-factors *R* are based on *F*, with *F* set to zero for negative *F*^2^. The threshold expression of *F*^2^ > σ(*F*^2^) is used only for calculating *R*-factors(gt) *etc*. and is not relevant to the choice of reflections for refinement. *R*-factors based on *F*^2^ are statistically about twice as large as those based on *F*, and *R*-factors based on ALL data will be even larger.

### Fractional atomic coordinates and isotropic or equivalent isotropic displacement parameters (Å^2^)

**Table:**

	*x*	*y*	*z*	*U*_iso_*/*U*_eq_	
C1	0.1978 (2)	1.3725 (3)	0.0997 (2)	0.0624 (7)	
H1	0.1549	1.4428	0.1392	0.075*	
C2	0.1992 (2)	1.3747 (3)	−0.0114 (3)	0.0623 (7)	
C3	0.2606 (2)	1.2750 (3)	−0.0732 (2)	0.0592 (6)	
H3	0.2605	1.2816	−0.1490	0.071*	
C4	0.32327 (19)	1.1634 (2)	−0.01984 (19)	0.0518 (6)	
H4	0.3655	1.0940	−0.0607	0.062*	
C5	0.32435 (18)	1.1529 (2)	0.09343 (18)	0.0471 (6)	
C6	0.26236 (18)	1.2618 (2)	0.1518 (2)	0.0564 (6)	
H6	0.2645	1.2601	0.2277	0.068*	
C7	0.39214 (17)	1.0387 (2)	0.15185 (19)	0.0495 (5)	
H7	0.4307	1.0764	0.2121	0.059*	
C8	0.40628 (16)	0.8869 (2)	0.13044 (17)	0.0466 (5)	
C9	0.4813 (2)	0.8002 (3)	0.20544 (19)	0.0529 (6)	
C10	0.4617 (2)	0.6306 (3)	0.2298 (2)	0.0654 (7)	
H10A	0.3849	0.6052	0.2133	0.078*	
H10B	0.4735	0.6124	0.3071	0.078*	
C11	0.5378 (2)	0.5233 (3)	0.1644 (2)	0.0694 (8)	
H11A	0.6138	0.5581	0.1745	0.083*	
H11B	0.5323	0.4197	0.1951	0.083*	
C12	0.5154 (2)	0.5126 (3)	0.0430 (2)	0.0627 (6)	
H12A	0.4396	0.4768	0.0327	0.075*	
H12B	0.5643	0.4345	0.0122	0.075*	
C13	0.53068 (18)	0.6639 (3)	−0.0216 (2)	0.0565 (6)	
H13A	0.5763	0.7339	0.0214	0.068*	
H13B	0.5715	0.6403	−0.0879	0.068*	
C14	0.42430 (19)	0.7491 (3)	−0.05315 (19)	0.0586 (6)	
H14A	0.3820	0.6829	−0.1020	0.070*	
H14B	0.4446	0.8417	−0.0937	0.070*	
C15	0.34864 (16)	0.7973 (2)	0.0413 (2)	0.0534 (5)	
H15A	0.2886	0.8604	0.0125	0.064*	
H15B	0.3159	0.7046	0.0728	0.064*	
F1	0.13754 (15)	1.48509 (16)	−0.06379 (17)	0.0991 (5)	
O1	0.56001 (15)	0.86558 (19)	0.24861 (14)	0.0704 (5)	

### Atomic displacement parameters (Å^2^)

**Table:**

	*U*^11^	*U*^22^	*U*^33^	*U*^12^	*U*^13^	*U*^23^
C1	0.0563 (14)	0.0496 (14)	0.081 (2)	0.0065 (11)	0.0107 (15)	−0.0091 (14)
C2	0.0545 (14)	0.0467 (14)	0.086 (2)	0.0093 (12)	−0.0095 (14)	0.0034 (13)
C3	0.0650 (16)	0.0544 (13)	0.0583 (15)	0.0011 (12)	−0.0012 (12)	0.0033 (13)
C4	0.0543 (14)	0.0440 (12)	0.0572 (15)	0.0045 (10)	0.0064 (12)	−0.0006 (11)
C5	0.0503 (14)	0.0413 (12)	0.0499 (14)	0.0000 (9)	0.0058 (12)	−0.0013 (10)
C6	0.0613 (15)	0.0497 (12)	0.0583 (14)	−0.0031 (11)	0.0086 (13)	−0.0042 (13)
C7	0.0552 (13)	0.0497 (11)	0.0437 (12)	−0.0012 (10)	0.0040 (11)	0.0004 (11)
C8	0.0446 (12)	0.0456 (11)	0.0494 (14)	−0.0003 (9)	0.0051 (11)	0.0017 (10)
C9	0.0619 (15)	0.0542 (13)	0.0426 (13)	0.0023 (12)	0.0037 (12)	0.0032 (11)
C10	0.0829 (17)	0.0545 (14)	0.0587 (16)	0.0075 (13)	0.0069 (14)	0.0140 (12)
C11	0.0798 (19)	0.0520 (13)	0.077 (2)	0.0113 (12)	−0.0010 (16)	0.0103 (13)
C12	0.0629 (14)	0.0498 (13)	0.0754 (17)	0.0080 (10)	−0.0007 (14)	−0.0058 (14)
C13	0.0572 (13)	0.0571 (13)	0.0551 (14)	0.0038 (10)	0.0045 (11)	−0.0084 (11)
C14	0.0651 (15)	0.0564 (13)	0.0543 (14)	−0.0007 (11)	−0.0112 (13)	−0.0075 (11)
C15	0.0454 (11)	0.0466 (11)	0.0682 (14)	0.0005 (9)	−0.0094 (13)	0.0010 (12)
F1	0.1013 (12)	0.0788 (9)	0.1173 (13)	0.0376 (9)	−0.0210 (11)	0.0041 (9)
O1	0.0841 (12)	0.0686 (10)	0.0583 (11)	−0.0009 (9)	−0.0200 (10)	0.0053 (8)

### Geometric parameters (Å, °)

**Table:**

C1—C2	1.361 (4)	C10—C11	1.527 (3)
C1—C6	1.385 (3)	C10—H10A	0.9700
C1—H1	0.9300	C10—H10B	0.9700
C2—C3	1.361 (3)	C11—C12	1.514 (4)
C2—F1	1.365 (3)	C11—H11A	0.9700
C3—C4	1.384 (3)	C11—H11B	0.9700
C3—H3	0.9300	C12—C13	1.535 (3)
C4—C5	1.390 (3)	C12—H12A	0.9700
C4—H4	0.9300	C12—H12B	0.9700
C5—C6	1.395 (3)	C13—C14	1.524 (3)
C5—C7	1.464 (3)	C13—H13A	0.9700
C6—H6	0.9300	C13—H13B	0.9700
C7—C8	1.343 (3)	C14—C15	1.529 (3)
C7—H7	0.9300	C14—H14A	0.9700
C8—C9	1.488 (3)	C14—H14B	0.9700
C8—C15	1.506 (3)	C15—H15A	0.9700
C9—O1	1.222 (3)	C15—H15B	0.9700
C9—C10	1.508 (3)		
			
C2—C1—C6	117.6 (2)	H10A—C10—H10B	107.8
C2—C1—H1	121.2	C12—C11—C10	116.4 (2)
C6—C1—H1	121.2	C12—C11—H11A	108.2
C1—C2—C3	123.6 (2)	C10—C11—H11A	108.2
C1—C2—F1	118.1 (3)	C12—C11—H11B	108.2
C3—C2—F1	118.2 (3)	C10—C11—H11B	108.2
C2—C3—C4	118.1 (2)	H11A—C11—H11B	107.3
C2—C3—H3	121.0	C11—C12—C13	115.7 (2)
C4—C3—H3	121.0	C11—C12—H12A	108.4
C3—C4—C5	121.4 (2)	C13—C12—H12A	108.4
C3—C4—H4	119.3	C11—C12—H12B	108.4
C5—C4—H4	119.3	C13—C12—H12B	108.4
C4—C5—C6	117.6 (2)	H12A—C12—H12B	107.4
C4—C5—C7	122.4 (2)	C14—C13—C12	115.95 (19)
C6—C5—C7	119.9 (2)	C14—C13—H13A	108.3
C1—C6—C5	121.7 (3)	C12—C13—H13A	108.3
C1—C6—H6	119.1	C14—C13—H13B	108.3
C5—C6—H6	119.1	C12—C13—H13B	108.3
C8—C7—C5	128.9 (2)	H13A—C13—H13B	107.4
C8—C7—H7	115.5	C13—C14—C15	116.02 (19)
C5—C7—H7	115.5	C13—C14—H14A	108.3
C7—C8—C9	116.4 (2)	C15—C14—H14A	108.3
C7—C8—C15	125.55 (19)	C13—C14—H14B	108.3
C9—C8—C15	118.01 (17)	C15—C14—H14B	108.3
O1—C9—C8	120.40 (19)	H14A—C14—H14B	107.4
O1—C9—C10	118.8 (2)	C8—C15—C14	114.40 (16)
C8—C9—C10	120.8 (2)	C8—C15—H15A	108.7
C9—C10—C11	112.8 (2)	C14—C15—H15A	108.7
C9—C10—H10A	109.0	C8—C15—H15B	108.7
C11—C10—H10A	109.0	C14—C15—H15B	108.7
C9—C10—H10B	109.0	H15A—C15—H15B	107.6
C11—C10—H10B	109.0		
			
C6—C1—C2—C3	−0.2 (4)	C7—C8—C9—O1	−31.5 (3)
C6—C1—C2—F1	−178.79 (18)	C15—C8—C9—O1	151.4 (2)
C1—C2—C3—C4	1.3 (4)	C7—C8—C9—C10	148.4 (2)
F1—C2—C3—C4	179.8 (2)	C15—C8—C9—C10	−28.7 (3)
C2—C3—C4—C5	−0.2 (4)	O1—C9—C10—C11	−79.6 (3)
C3—C4—C5—C6	−1.8 (4)	C8—C9—C10—C11	100.6 (3)
C3—C4—C5—C7	−178.35 (18)	C9—C10—C11—C12	−70.2 (3)
C2—C1—C6—C5	−1.9 (4)	C10—C11—C12—C13	63.3 (3)
C4—C5—C6—C1	2.9 (3)	C11—C12—C13—C14	−102.8 (3)
C7—C5—C6—C1	179.6 (2)	C12—C13—C14—C15	58.4 (3)
C4—C5—C7—C8	−44.6 (4)	C7—C8—C15—C14	109.4 (2)
C6—C5—C7—C8	138.9 (2)	C9—C8—C15—C14	−73.8 (2)
C5—C7—C8—C9	180.0 (2)	C13—C14—C15—C8	51.9 (3)
C5—C7—C8—C15	−3.2 (4)		
